# Insights into the Lysine Acetylome of the Haloarchaeon *Haloferax volcanii* during Oxidative Stress by Quantitative SILAC-Based Proteomics

**DOI:** 10.3390/antiox12061203

**Published:** 2023-06-01

**Authors:** Ricardo L. Couto-Rodríguez, Jin Koh, Sixue Chen, Julie A. Maupin-Furlow

**Affiliations:** 1 Department of Microbiology and Cell Science, Institute of Food and Agricultural Sciences, University of Florida, Gainesville, FL 32611, USA; r.coutorodriguez@ufl.edu; 2 Proteomics and Mass Spectrometry, Interdisciplinary Center for Biotechnology Research, University of Florida, Gainesville, FL 32610, USA; jinkoh@ufl.edu (J.K.); schen8@olemiss.edu (S.C.); 3 Genetics Institute, University of Florida, Gainesville, FL 32610, USA; 4 Department of Biology, University of Florida, Gainesville, FL 32611, USA; 5 Department of Biology, The University of Mississippi, Oxford, MS 38677, USA

**Keywords:** *Archaea*, halophiles, oxidative stress, mass spectrometry, SILAC, lysine acetylation

## Abstract

Oxidative stress adaptation strategies are important to cell function and are linked to cardiac, neurodegenerative disease, and cancer. Representatives of the *Archaea* domain are used as model organisms based on their extreme tolerance to oxidants and close evolutionary relationship with eukaryotes. A study of the halophilic archaeon *Haloferax volcanii* reveals lysine acetylation to be associated with oxidative stress responses. The strong oxidant hypochlorite: (i) stimulates an increase in lysine acetyltransferase *Hv*Pat2 to *Hv*Pat1 abundance ratios and (ii) selects for lysine deacetylase *sir2* mutants. Here we report the dynamic occupancy of the lysine acetylome of glycerol-grown *H. volcanii* as it shifts in profile in response to hypochlorite. These findings are revealed by the: (1) quantitative multiplex proteomics of the SILAC-compatible parent and *Δsir2* mutant strains and (2) label-free proteomics of H26 ‘wild type’ cells. The results show that lysine acetylation is associated with key biological processes including DNA topology, central metabolism, cobalamin biosynthesis, and translation. Lysine acetylation targets are found conserved across species. Moreover, lysine residues modified by acetylation and ubiquitin-like sampylation are identified suggesting post-translational modification (PTM) crosstalk. Overall, the results of this study expand the current knowledge of lysine acetylation in *Archaea*, with the long-term goal to provide a balanced evolutionary perspective of PTM systems in living organisms.

## 1. Introduction

Oxidative stress and post-translational response mechanisms are of interest in biological applications due to their association with diseases such as neurodegenerative disorders, heart failure, and cancer [1,2,3,4]. *Archaea* are used as model organisms because of their close evolutionary relationship and similar information-processing mechanisms with eukaryotes [5,6]. Halophilic *archaea*, such as *Haloferax volcanii*, thrive in hypersaline environments and commonly encounter oxidative stress due to the presence of high salt, high UV radiation, and desiccation in these ecosystems [7]. Oxidizing conditions lead to the formation of reactive oxygen species (ROS) that can damage proteins, lipids, carbohydrates, and nucleic acids [8]. Hypochlorous acid (HOCl) and its dissociated derivative hypochlorite (ClO^−^) are oxidizers prevalent in hypersaline environments, where NaCl concentrations exceed 35 g/L and chloride ions are prevalent [9]. To overcome these extreme conditions, halophilic organisms such as *H. volcanii* have adopted several strategies such as ROS scavenging by carotenoid pigments, ROS detoxification via superoxide dismutase, and DNA repair mechanisms related to eukaryotic homologous recombination and bacterial UvrABCD systems [10,11].

Proteomic response to oxidative stress in *H. volcanii* was recently assessed via a multiplex quantitative approach that used stable isotope labeling of amino acids in cell culture (SILAC) followed by multidimensional liquid chromatography with tandem mass spectrometry (MDLC-MS/MS) [12]. Several alterations of the proteome were observed including a differential abundance of proteins related to sulfur mobilization, lipid biosynthesis, DNA repair, and post-translational modification (PTM) systems [12]. Protein lysine acetylation, a PTM commonly used in regulation [13], was suggested to have a role in response to oxidative damage based on finding the protein abundance ratios of two lysine acetyltransferase (KAT) homologs, *Hv*Pat2 to *Hv*Pat1, increased six-fold during hypochlorite exposure [12].

While thoroughly studied in eukaryotes and bacteria, lysine acetylation in *archaea* is less commonly reported. Most of the current knowledge of this modification in *archaea* stems from *Sulfolobus solfataricus* where chromatin remodeling protein Alba is found in lysine acetylated by Pat and deacetylated by Sir2 [14,15]. Similarly, reports of lysine acetylome profiling of *archaea* by MS analysis are few. LC-MS/MS analysis of the *Haloferax mediterranei* lysine acetylome after immunoaffinity enrichment revealed 17% of the proteome to be lysine acetylated including key enzymes in replication, carbon storage, central metabolism, and protein biosynthesis, suggesting a prominent role of lysine acetylation in these processes [16]. Similarly analysis of the *Sulfolobus islandicus* lysine acetylome that included tandem mass tag (TMT)-based quantification and immunoaffinity enrichment provided a global perspective on the lysine acetylome and guided the detailed analysis of six acyl-CoA synthetases as Pat substrates [17]. Immunocapture of lysine acetylated (Kac) peptides coupled with LC-MS/MS analysis has also provided a road map for understanding the lysine acetylome of *Thermococcus gammatolerans* [18].

Even though the function of lysine acetylation has been extensively explored in eukaryotes and bacteria, more knowledge is needed in *archaea*. In particular, a global perspective on how the archaeal lysine acetylome may respond to environmental cues is not yet known. In this present study, we aim to define the role of lysine acetylation during oxidative stress response in *H. volcanii*. The rationale for this focus is based on our previous finding that oxidative stress: (i) stimulates an increase in the abundance ratios of the lysine acetyltransferase homologs *Hv*Pat2 to *Hv*Pat1 and (ii) selects for the survival of a *sir2* transposon mutant [12,19]. Here a quantitative comparison of the lysine acetylome is presented during the presence vs. absence of hypochlorite stress. SILAC-based and label-free approaches were coupled with LC-MS/MS analysis to provide a detailed roadmap of the lysine acetylome and to determine shifts in the occupancy of the lysine acetylation (Kac) sites when compared to the total proteome. Two strains were used for this analysis: (i) LM08 (H26 *∆lysA∆argH*), a lysine and arginine auxotroph shown to incorporate “light” or “heavy” lysine and arginine amino acids for SILAC analysis [12], and (ii) RC04 (LM08 *∆sir2*), a derivative of LM08 that has the lysine deacetylase *sir2* gene deleted to stabilize and enhance detection of the acetylome. The lysine acetylome of the ‘wild type’ H26 was also analyzed using a label-free approach. The results of the study reveal hypochlorite stress and the *sir2* mutation alter the occupancy of Kac sites and protein abundance. The Kac targets were found enriched in pathways associated with DNA topology, central metabolism, and translation. The combined use of SILAC and label-free approaches was found to enhance the lysine acetylome coverage. Overall, this study advances the knowledge of lysine acetylation and oxidative stress response in halophilic *archaea*.

## 2. Materials and Methods

### 2.1. Materials

Biochemicals and analytical-grade inorganic chemicals were purchased from Fisher Scientific (Atlanta, GA, USA), Bio-Rad (Hercules, CA, USA), and Sigma-Aldrich (St. Louis, MO, USA). Oligonucleotides were purchased from Eurofins Genomics (Louisville, KY, USA). DNA polymerases and restriction enzymes were from New England Biolabs (Ipswich, MA, USA). GeneRuler 1 kb plus DNA standards were from ThermoFisher (Waltham, MA, USA). Sodium hypochlorite (NaOCl) was reagent grade (available chlorine 10–15%, Sigma-Aldrich, #425044-250 mL). Amino acid isotopes were from Cambridge Isotope Laboratories, Inc. (Tewksbury, MA, USA).

### 2.2. Strains, Media, and Growth Conditions

Details of strains, plasmids, and primer sequences used are listed in Table 1. *E. coli* cultures were grown at 37 °C in Luria–Bertani (LB) medium supplemented with ampicillin (100 μg·mL^−1^). *H. volcanii* strains were grown at 42 °C in ATCC974 complex medium or glycerol minimal medium (GMM) as described previously [12]. *H. volcanii* media was supplemented as needed with novobiocin (0.2 μg·mL^−1^), (light) L-lysine (+0) (0.3 mM), (light) L-arginine (+0) (0.3 mM), (heavy) L-lysine (+8) (0.3 mM), and L-arginine (+6) (0.3 mM). Liquid cultures were grown with rotary shaking at 200 rpm. Culture plates were supplemented with 1.5% (*w*/*v*) agar. Growth was measured by optical density at 600 nm (OD_600_) where 1 OD_600_ unit equals approximately 10^9^ CFU·mL^−1^.

### 2.3. Generation of Mutant Strains

*H. volcanii* JM503 (H26 *∆sir2*) mutant was generated via the *pyrE2*-based pop-in/pop-out deletion method [22]. Pre-knockout plasmid pJAM4011 was generated by ligation of DNA fragments containing the *sir2* gene and 5′ and 3′ flanking regions (500 bp each) into the BamHI and KpnI sites of plasmid pTA131. The DNA region for pJAM4011 was amplified using primer pair 9/10 and genomic DNA from *H. volcanii* H26 as a template. Knock-out plasmid pJAM4015 was constructed via inverse PCR using primer pair 11/12 and plasmid pJAM4011 as templates. Primer pairs 9/10 and 13/14 were used to screen for deletion mutants. A similar strategy was used to generate *H. volcanii* strain RC04. For *H. volcanii* strain JM506, pre-knockout plasmids pJAM4009 and 4010 were generated using primer pairs 1/2 and 3/4 respectively. Knockout plasmids pJAM4013 and pJAM4014 were constructed via inverse PCR using primer pair 5/6 and 7/8, respectively, with pJAM 4009 and 4010 as templates. Primer pairs 1/2 and 3/4 were used to screen for deletion mutants.

### 2.4. Immunoblotting Analysis of the Lysine Acetylome of H. volcanii Cells

*H. volcanii* cells were streaked with a toothpick from 20% (*v*/*v*) glycerol stocks stored at −80 °C onto ATCC974 plates and grown for 3–4 days at 42 °C. Cells from isolate colonies were inoculated into 2 mL of GMM in 13 × 100 mm culture tubes and grown for 3 days (200 rpm, 42 °C). Cells from this culture were subcultured to a final OD_600_ of 0.02 into 2 mL GMM medium and grown overnight (200 rpm, 42 °C). Cells were transferred in duplicate into 50 mL GMM medium (in a 250 mL Erlenmeyer flask) and grown overnight (200 rpm, 42 °C) to an OD_600_ of 0.4–0.6. Cultures were exposed to 3 mM NaOCl for 20 min at 42 °C with orbital shaking (200 rpm) and immediately harvested (1 mL) by centrifugation (16,100× *g* for 10 min at room temperature). For the time course, samples were exposed for 5, 15, 30, 45, and 60 min at 42 °C. The spent medium was completely removed, and resulting cell pellets were immediately frozen at −80 °C until use. Cell pellets were thawed on ice and resuspended to 0.01 OD_600_ unit per μL of reducing Laemmli Sample Buffer (LSB) with 10% (*v*/*v*) β-mercaptoethanol. Samples were boiled for 10 min and applied at 4 μL (0.04 OD_600_ units of cells) per 12% SDS-PAGE gel. After electrophoresis, proteins were electroblotted from unstained SDS-PAGE gels onto a 0.45 μm polyvinylidene difluoride (PVDF) membrane (MilliporeSigma, Burlington, MA, USA) at 4 °C for 13 h at 30 V using mini trans-blot module according to supplier’s instructions (Bio-Rad). The membrane was placed upright in an 18 × 10 cm plastic container and briefly rinsed with 25 mL of 1X TBS (50 mM Tris-Cl, pH 7.5, 150 mM NaCl). Blocking was performed for 3 h at 4 °C in 25 mL TBS supplemented with 0.1% (*v*/*v*) Tween 20 and 5% (*w*/*v*) bovine serum albumin (BSA) as recommended by the supplier. Primary antibody (cat # PTM-105, Pan anti-acetyllysine polyclonal antibody in rabbit; PTM Bio, Chicago, IL, USA) was added at 1:5000 dilution to 25 mL of fresh blocking buffer and incubated with the membrane for 1 h at room temperature with medium speed rocking. The membrane was washed 5 times in 50 mL TBS supplemented with 0.1% (*v*/*v*) Tween 20 (TBST) for 5 min per wash using the high setting of the rocker. A secondary antibody of mouse anti-rabbit IgG (whole molecule)-HRP-linked antibody (SC-2357, lot #A0318; 200 ugs per 0.5 mL; Santa Cruz Biotech, Dallas, TX, USA) was diluted 1:12,500 in 25 mL of blocking solution and incubated with the membrane for 1 h with medium rocking. The membrane was washed 5 times in 50 mL TBS supplemented with 0.1% (*v*/*v*) Tween 20 (TBST) for 5 min per wash using the high setting of the rocker. The antibody:protein complexes were visualized on the PVDF membrane using ECL Prime substrate (Cytiva, Marlborough, MA, USA) according to the supplier’s guidelines. Membranes were imaged using X-ray film.

### 2.5. Hypochlorite Exposure for Mass Spectrometry Analysis

*H. volcanii* LM08 and RC04 were streaked with a toothpick from 20% (*v*/*v*) glycerol stocks stored at −80 °C onto a plate of GMM supplemented with 0.3 mM lysine and 0.3 mM arginine and incubated at 42 °C for 5 days. Isolated colonies (four biological replicates per strain, *n* = 4) were inoculated to 25 mL GMM in 250 mL Erlenmeyer flasks supplemented with “heavy” or “light” amino acids and allowed to grow for 24 h. Cultures were subcultured to a final OD_600_ of 0.01 into 25 mL GMM in 250 mL Erlenmeyer flasks, and growth on the heavy or light medium was repeated for 24 h. Cells were again subcultured to a final OD_600_ of 0.01 in 25 mL of heavy or light medium until the late log phase (0.7–0.8). Cells were incubated for 20 min with rotary shaking (200 rpm) at 42 °C with 2.5 mM NaOCl (treatment groups) or 0 mM (mock H_2_O treatment) (control groups). For all replicates, the control group cultures were labeled with (heavy) L-lysine (+8) and L-arginine (+6), and the treatment group was labeled with (light) L-lysine (+0) and L-arginine (+0). Culture volumes were normalized in each centrifuge tube to equivalent total OD units per sample, and cells were harvested by centrifugation (4000× *g* at 25 °C for 30 min) and the supernatant was subsequently removed. Cell pellets of control and treatment groups were mixed at 1:1 ratio (*n* = 4 mixed samples) and the proteins were stored at −80 °C until further use.

### 2.6. TriZOL Extraction and Trypsin Digestion

Proteins were extracted from pellets using TRIzol as described by Kirkland et al. [23]. The resulting TRIzol extracted pellets were frozen at −80 °C until use. Processing of samples and LC-MS/MS analysis was performed as detailed by McMillan et al. [12]. Enrichment of acetylated peptides was performed using PTMScan^®^ HS Acetyl-Lysine Motif (Ac-K) (Cat No. 46784, Cell Signaling Technologies, Danvers, MA, USA) according to manufacturer’s instructions and subjected to LC-MS/MS analysis. 

SILAC-labeled protein pellets were dissolved in protein buffer (9.0 M urea, 20 mM HEPES (pH 8.0), 2.5 mM sodium pyrophosphate, 1 mM β-glycerol phosphate, 1 mM sodium orthovanadate) freshly supplemented with 10 mM sodium butyrate. Protein samples were quantified by the EZQ™ Protein Quantification Kit (Thermo Fisher Scientific, Eugene, OR, USA) with the SoftMax Pro Software v5.3 under the SpectraMax M5 (Molecular Devices, LLC., San Jose, CA, USA). Proteins were reduced with the final concentration of 7.5 mM of Tris-2-carboxyethyl phosphine (TCEP; Thermo Fisher Scientific) at 60 °C for 60 min and subsequently alkylated with the final concentration of 15 mM of methyl methanethiosulfonate (MMTS; Thermo Fisher Scientific) at room temperature for 30 min in the dark. Prior to digestion, the urea concentration was reduced to 1 M with 20 mM HEPES (pH 8.0), and protein samples were digested with LysC/trypsin (Thermo Fisher Scientific; 1:100, protease/protein mass) overnight at 37 °C. Samples were acidified with the final concentration of 0.1% formic acid (*v*/*v*) and desalted on an Oasis HLB 1 cc Vac Cartridge (30 mg Sorbent; Waters Milford, MA, USA). 

Immunoaffinity enrichment of lysine acetylated peptides from *H. volcanii* was performed using the PTMScan protocol according to the manufacturer’s instructions. Briefly, a total of 3 mg of lyophilized tryptic peptides was dissolved in 1.4 mL of immunoaffinity purification (IAP) buffer (50 mM MOPS, pH 7.2, 10 mM sodium phosphate, 50 mM NaCl), and the lysine acetylation antibody (Cell Signaling Technology, Danvers, MA, USA) was washed with 1 mL of PBS. The supernatant of tryptic peptides was transferred into the lysine acetylation antibody overnight at a rotator at 4 °C for the conjugation. The beads were washed twice with 1 mL of IAP buffer and three times with 1 mL of chilled HPLC grade water (Burdick and Jackson, Muskegon, MI, USA) three times. Peptides were eluted from beads with 55 μL of 0.15% of trifluoroacetic acid (TFA) standing for 10 min at room temperature and sequentially followed by the elution with 50 μL of 0.15% TFA. The eluted samples were desalted with C18-Ziptip (Millipore, Billerica, MA, USA). 

### 2.7. Data Acquisition with Data Dependent Decision Tree

An Orbitrap Fusion Tribrid Mass Spectrometer system (Thermo Fisher Scientific, San Jose, CA, USA) was interfaced with an ultra-performance Easy-nLC 1200 system (Thermo Fisher Scientific, Bremen, Germany). Each desalted sample was loaded onto an Acclaim Pepmap 100 pre-column (20 mm × 75 μm; 3 μm-C18) and eluted using a PepMap RSLC analytical column (500 mm × 75 μm; 2 μm-C18). The flow rate was set at 250 nl/min with solvent A (0.1% formic acid, 99.9% water (*v*/*v*)) and solvent B (0.1% formic acid, 80% acetonitrile, 19.9% water (*v*/*v*)) as the mobile phases followed by a linear gradient: 1–2% of solvent B in 5 min, 2–40% of solvent B in 95 min, ramping up to 98% solvent B in 2 min, and isocratic at 98% in 13 min. 

The mass spectrometer acquired the data under the collision ion dissociation (CID) mode in each MS and MS/MS cycle scanning from 350 to 1800 m/z. The maximum ion injection times for the MS scan and the MS/MS scans were 35 ms. MS1 spectra were recorded at resolution at 120,000 FWHM from 350–1800 *m*/*z* with a quadrupole isolation window of 1.3. The number of selected MS1 ions for fragmentation was determined by the Top Speed acquisition algorithm and a dynamic exclusion of 60 s. The MS/MS ions were separated in the linear ion trap with CID fragmentation (Rapid; NCE 35%; maximum injection time 35 ms; AGC 1 × 10^4^). The normalized collision energy (NCE) was set to 35% for each fragmentation method and one microscan was acquired for each spectrum. The automated gain control (AGC) target was set to 2 × 10^5^, with a max. injection time of 50 ms. Only precursors with charge states 2–7 with an intensity higher than 1 × 10^4^ were selected for fragmentation. The monoisotopic precursor selection (MIPS) filter was activated. The option to inject ions for all available parallelizable times was selected. 

### 2.8. Data Analysis

RAW mass spectrometry files were processed using MaxQuant (v.2.2.0.0) with an integrated Andromeda search engine. Tandem mass spectra were searched against the Uniprot *H. volcanii* DS2 database (Accession: UP000320212) with 3996 sequences concatenated with a reverse decoy database. The mass tolerance for precursor ions was set at 20 ppm in the first search and 5 ppm in the main search and the mass tolerance for fragment ions was set at 0.8 Da. Trypsin/P was specified as the cleavage enzyme allowing up to 2 missed cleavages. The false discovery rate was set at 1% and the minimum peptide length was set at 7. Static modification of methylthio was allowed on all cysteines while dynamic modifications of acetyl (N-terminal and K-acetyl), and methionine oxidation were used. Quantitative information for modification was determined according to SILAC (light:heavy) ratios. The false discovery rate for quantified proteins was set at <0.01, a differentially regulated protein was log2 transformed, and then determined at a 1.2-fold cut-off and Adj *p*-value < 0.05. The false discovery rate for modified peptides was adjusted to <0.01 and the minimum score for modified peptides was set at >40. Quantitative information for each peptide or lysine site was quantified according to SILAC (light:heavy) ratios. The resulting evidence files were subject to further analysis using the Proteus R package for statistical analysis [24]. Mean Log2 transformed ratios were used to calculate a moderate t-statistic in order to determine a *p*-value for each protein or acetylated peptide which was then adjusted for multiple hypotheses testing using the Benjamini–Hochberg method with a false discovery rate of ≤0.1 to obtain Adj. *p*-value. To correct for changes in protein expression, the mean log2 ratios of corresponding proteins were subtracted from the log2 ratios of each acetyl peptide. Differentially acetylated peptides were identified at a 1.2-cut-off and Adj *p*-value < 0.05. Interaction network enrichment was performed using STRING-DB ver. 11.5 [25]. Venn diagrams for comparison were rendered using Venny 2.0.2 (https://bioinfogp.cnb.csic.es/tools/venny/index.html [accessed on 27 January 2023]). 

## 3. Results

### 3.1. Exposure to Sodium Hypochlorite Stimulates an Increase in the Abundance of the Lysine Acetylome of H. volcanii

**Figure 1:**
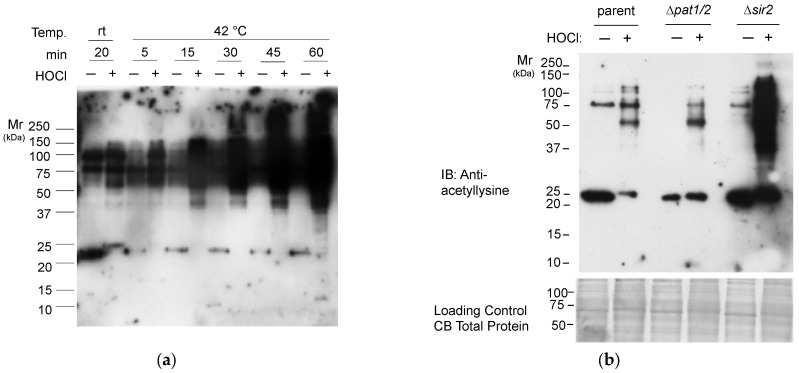
Hypochlorite stress leads to an increased abundance of lysine acetylation targets. (**a**). Lysine acetylation profile of parent strain (H26) after exposure to NaOCl. Parent was exposed to either a mock H_2_O control or 3 mM NaOCl for the time and temperature specified. (**b**) Lysine acetylation profile of parent (H26), *∆pat1∆pat2* (JM506) and *∆sir2* (JM503) strains. Cultures were exposed to either a mock H_2_O treatment or 3 mM NaOCl for 20 min at 25 °C. Proteins were separated by reducing SDS-PAGE and analyzed by immunoblotting using Pan-anti-acetyllysine antibodies (see Section 2 for details). Coomassie Blue loading controls were also run to ensure equal loading of proteins.

To determine how prevalent lysine acetylation is during oxidative stress, *H. volcanii* “wild type” cells were grown on glycerol minimal medium, exposed to sodium hypochlorite vs. a mock control, and then analyzed by immunoblotting using a pan anti-acetyllysine antibody (Figure 1a). This approach enabled the detection of shifts in the lysine acetylome of *H. volcanii* in response to hypochlorite as an environmental cue. When the cells were exposed to hypochlorite at room temperature or 42 °C, the lysine acetylome was found to increase in abundance over time (from 0 to 60 min). The banding pattern of the Kac proteins was also found to be altered by the sodium hypochlorite treatment, particularly in the 20–25 kDa range. The lysine acetylome responses of a *∆sir2* mutant and a *∆pat1∆pat2* mutant were also evaluated (Figure 1b). In the absence of *Hv*Pat1 and *Hv*Pat2, a modest reduction in the abundance of Kac targets was observed during hypochlorite stress. By contrast, a dramatic accumulation of Kac modifications was observed in the absence of Sir2 suggesting this sirtuin-type lysine deacetylase homolog is an important factor during oxidative stress response. This immunoblotting analysis establishes a link between hypochlorite stress and lysine acetylation with the abundance of Kac modifications stimulated during sodium hypochlorite exposure.

### 3.2. LC-MS/MS Analysis of ∆sir2 Mutant Reveals Dramatic Alterations in the Proteome

**Figure 2:**
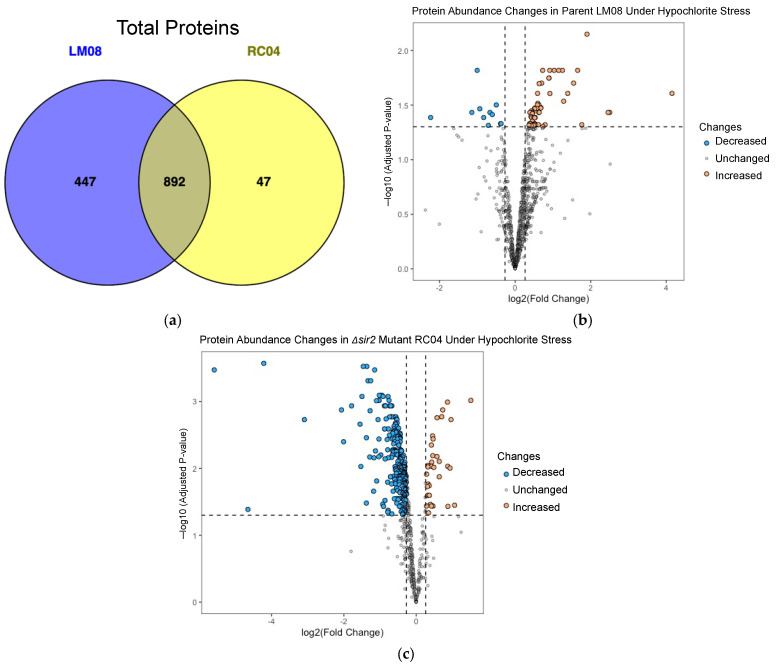
Comparison of identified total proteins between LM08 and RC04 strain. (**a**) Venn diagram of identified proteins across all samples with at least two peptides with an FDR adjusted q-value of 0.01 or less. (**b**) Volcano plot of quantified proteins in LM08. Abundance values are displayed as log_2_ of the fold-change ratio (NaOCl treatment compared to control) and −log_10_ of the adjusted *p*-value using the Benjamini-Hochberg method. Vertical dotted lines indicate up/downregulation and horizontal lines indicate statistical significance (Adj *p*-value < 0.05). (**c**) Volcano plot of quantified proteins in RC04. Abundance values are displayed as log_2_ of the fold-change ratio (NaOCl treatment compared to control) and −log_10_ of the adjusted *p*-value using the Benjamini-Hochberg method. Vertical dotted lines indicate up/downregulation and horizontal lines indicate statistical significance (Adj *p*-value < 0.05).

To stabilize proteins in the Kac state and to facilitate the identification of the lysine acetylome, a SILAC-compatible *∆sir2* mutant was constructed. This newly generated strain, RC04 (LM08 *∆sir2*), and its parent LM08 (H26 *∆lysA ∆argH*) were subsequently grown on glycerol minimal medium in the presence of ‘heavy’ or ‘light’ amino acids, exposed to sodium hypochlorite or a mock control, and then analyzed by SILAC-based proteomics. Peptides enriched using anti-acetyllysine antibodies and total protein were subjected to LC-MS/MS analysis. In total, 1339 proteins were identified in LM08 (*n* = 4) and 939 in RC04 (*n* = 4) (Figure 2a; Supplementary Table S1A,B), representing coverage of 35% and 26% of the theoretical proteome, respectively. Most of these proteins could be quantified using MaxQuant (1047 or 78% in LM08 and 627 or 67% in RC04). 

In LM08, 74 proteins were observed to be of differential abundance with 63 proteins found to increase and 11 proteins found to decrease after exposure to hypochlorite (Figure 2b). Similarly to our previous SILAC dataset in McMillan et al. [12], proteins found commonly increased in abundance were associated with sulfur relay/metabolism (TrxB1 HVO_1031, MetY2 HVO_2997), transcription (ArsR-type HVO_0163, Tfb1 HVO_1052, Tfb2 HVO_1676), metal transport (ZnuC2 HVO_A0610, HVO_A0627), universal stress signaling (HVO_2534), and DNA recombination/repair (HVO_0990, HVO_1690, Dna2 HVO_2767), once again highlighting the role of these pathways in the response to hypochlorite stress.

**Figure 3:**
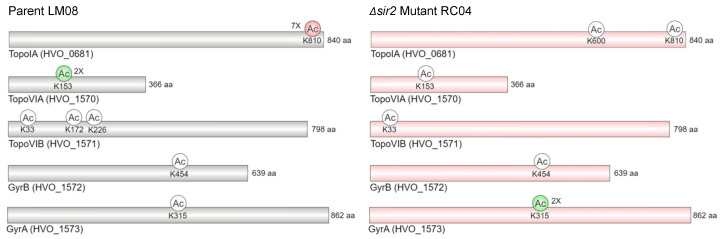
Hypochlorite induced changes in protein abundance and Kac occupancy of enzymes related to DNA topology in LM08 and RC04 (*Δsir2*) strains. Enzymes with schematic representation along with their Kac sites include: TopoIA—DNA topoisomerase 1, TopoVIA and B—type 2 DNA topoisomerase 6 subunits A and B, GyrA and B—DNA gyrase subunits A and B. Color of circles and rectangles denotes: Grey—no significant change in enzyme abundance and/or acetylation levels, red—decrease in enzyme abundance and/or acetylation levels and green—increase enzyme abundance and/or acetylation levels.

In the RC04 dataset, 286 proteins were found to be of differential abundance with 33 proteins increased and 253 proteins decreased during hypochlorite stress (Figure 2c). Thus, dramatic shifts in protein abundance were observed during hypochlorite stress for the RC04 strain with more proteins being downregulated when compared to LM08. Many of the proteins upregulated in abundance in LM08 during hypochlorite stress were downregulated in RC04, as exemplified by the sulfur relay/metabolism homologs TrxB1 (HVO_1031) and MetY2 (HVO_2997). To determine if there was a trend in the stress-induced downregulated proteins and to obtain insights on physiological impact, pathway enrichment analysis via STRING interaction networks was performed. Using this approach, a significant enrichment of the cobalamin biosynthetic process, division septum assembly, and tetrapyrrole biosynthesis among other pathways was detected among the 253 proteins decreased in RC04 during hypochlorite stress (Table 2, Supplementary Table S2). Most notably, a reduced abundance of all proteins involved in DNA topology (Figure 3) was observed in RC04, suggesting that increased lysine acetylation can affect DNA supercoiling responsible for regulating processes such as DNA replication and transcription [26]. Overall, the protein abundance shifts suggest a variety of pathways are impacted by the *Δsir2* mutation during hypochlorite stress including the cobalamin biosynthetic process, DNA topology, and sulfur relay.

### 3.3. Lysine Acetylation Enrichment Analysis Reveals DNA Topology, Metabolism and Translation to Be Regulated

**Figure 4:**
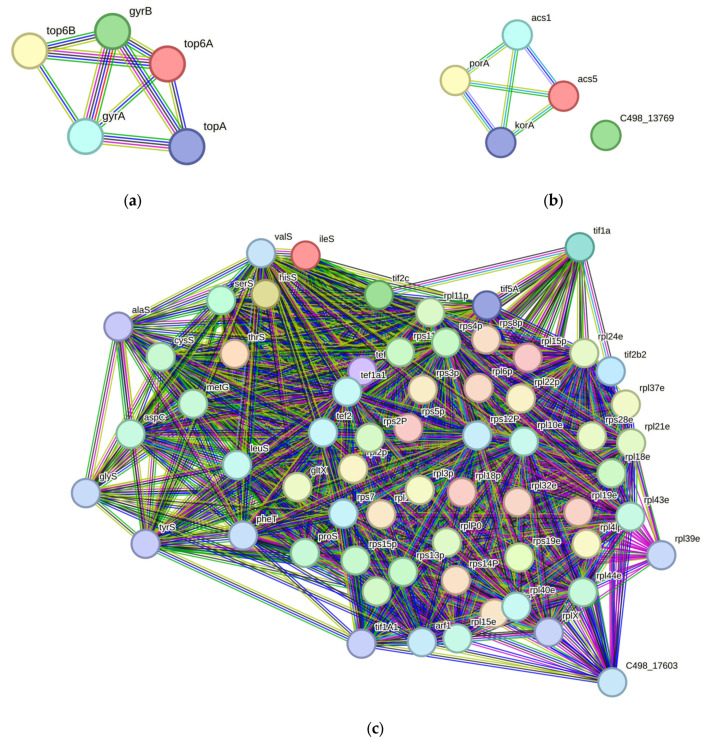
Enriched interaction networks of acetylated proteins by GO Biological Process via STRING-DB. (**a**) Subnetwork of acetylated proteins related to DNA topological change. (**b**) Subnetwork of acetylated proteins related to acetyl CoA biosynthesis. (**c**) Subnetwork of acetylated proteins related to translation and aminoacyl-tRNA synthesis.

To understand the impact of lysine acetylation and its role in the hypochlorite stress response, an immunoaffinity enrichment of lysine acetylated peptides using an anti-acetyllysine antibody was performed on the SILAC-labeled proteomes. In total, 686 Kac peptides representing 657 sites from 413 proteins in LM08, and 639 Kac peptides representing 611 sites from 343 proteins in RC04 were detected (Supplementary Table S3A,B) representing 30.8% and 36.5% of the total proteins detected respectively. When the pathway enrichment of lysine acetylated proteins was determined via STRING interaction networks (Supplementary Table S4A,B), a significant enrichment of DNA topological change was observed as the top hit in both strains (LM08 and RC04) with a network strength of 1.11 and 1.07, respectively. Upon further examination of the interaction network (Figure 4a), all 5 homologs predicted to control the superhelical torsion of the *H. volcanii* genomic DNA are observed to be modified by lysine acetylation. Other pathways found to be enriched in Kac proteins included acyl-CoA biosynthesis (Figure 4b) and translation including ribosomal proteins and aminoacyl synthetases (Figure 4c). 

**Figure 5:**
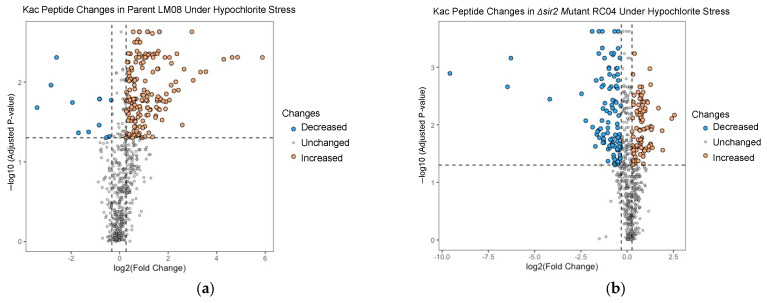
Lysine acetylation site changes after exposure to oxidative stress. (**a**) Volcano plot of quantified acetylated peptides in LM08. Abundance values are displayed as log_2_ of the fold-change ratio (NaOCl treatment compared to control) and −log_10_ of the adjusted *p*-value using the Benjamini-Hochberg method. Vertical dotted lines indicate up/downregulation and horizontal lines indicate statistical significance (Adj. *p*-value < 0.05). (**b**) Volcano plot of quantified acetylated peptides in RC04. Abundance values are displayed as log_2_ of the fold-change ratio (NaOCl treatment compared to control) and −log_10_ of the adjusted *p*-value using the Benjamini-Hochberg method. Vertical dotted lines indicate up/downregulation and horizontal lines indicate statistical significance (Adj. *p*-value < 0.05).

After correcting for changes in protein abundance and filtering for an adjusted *p*-value < 0.05, a total of 172 lysine residues were found altered in their acetylation state in LM08 and 192 in RC04 (Figure 5a,b; Supplementary Table S3A,B). These Kac sites, which were found of differential occupancy during hypochlorite stress, included 87 upregulated and 105 downregulated sites in RC04, and 163 upregulated and 9 downregulated sites in LM08. While these trends in Kac occupancy are in apparent contrast with the immunoblotting analysis, the following considerations should be noted: (i) Kac occupancy is distinct from Kac protein abundance, (ii) SILAC-based proteomics is based on relative quantification at the individual protein level, while immunoblotting analysis detects gross/overall changes in the proteome (portion which can be separated by SDS-PAGE and transferred to a PVDF membrane), (iii) proteolytic degradation can occur during mass spectrometry sample processing, whereas immunoblotting is an immediate readout, and (iv) lysine and/or arginine auxotrophy may have altered the response. 

When further comparing these two strains (RC04 and LM08) for hypochlorite-induced shifts in lysine acetylation the following is noted. For enzymes related to DNA topology, the acetylated state of K810 of TopoIA (HVO_0681) and K153 of TopoVIA (HVO_1570) were found at a 2-fold increase and 7-fold decrease in LM08, respectively (Figure 3). By contrast, a 2-fold increase in the lysine acetylated state of GyrA (HVO_1573) K315 was detected in RC04. Other notable differences during hypochlorite stress included finding in LM08 a decreased acetylation of K914 of HVO_0869 (a glutamate synthase (ferredoxin) (EC 1.4.7.1) homolog), K114 of a predicted Cro/C1 family transcription regulator (HVO_1299) and, although found in less than 3 replicates, K102 in the ATP-dependent Lon protease. By contrast, in RC04, the glutamate synthase (ferredoxin) K914, Cro/C1 family protein K114, and Lon protease K102 were unchanged in acetylation levels after exposure to hypochlorite.

### 3.4. Label Free Quantification Reveals a Greater Number of Lysine Acetylation Sites during Oxidative Stress in H. volcanii

**Figure 6:**
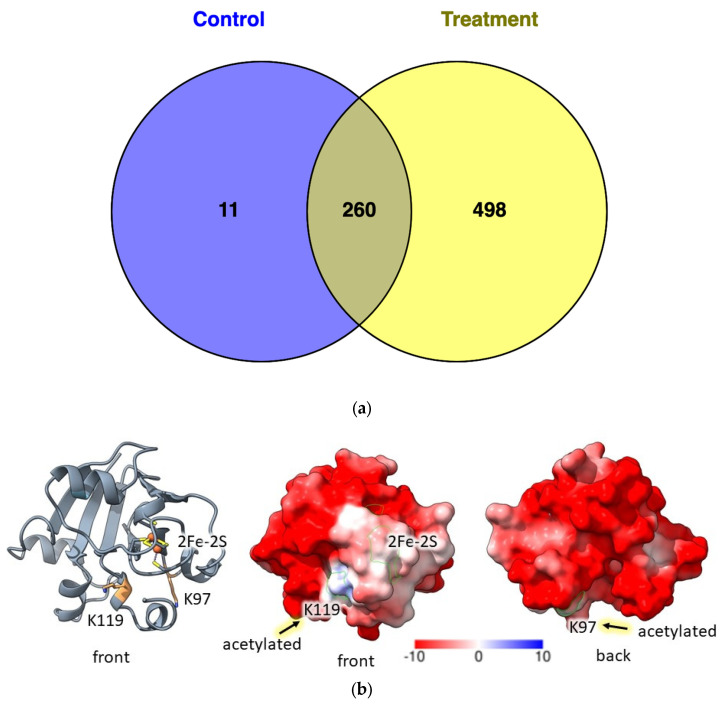
Label free analysis of acetylated proteins in parent strain (H26) after exposure to oxidative stress. (**a**) Venn diagram of acetylated proteins identified in WT during absence/presence of oxidative stress. (**b**) Model of HVO_2995 (Fdx) protein using Phyre2 based homology with acetylated lysine residue as well as predicted iron sulfur cluster cysteines highlighted in yellow (**left**). Surface model of Fdx protein. Positive/neutral charges only on acetylation site suggesting possible site for protein interaction (**middle** and **right**).

To obtain a comprehensive perspective of the Kac sites that occur in the presence and absence of oxidative stress, a label-free quantitative (LFQ) analysis of the H26 parent was also performed. After exposure to sodium hypochlorite or mock treatment (4 biological replicates for each condition), replicates were pooled and subjected to immunoaffinity enrichment and LC-MS/MS of Kac peptides. Using this method, 1632 Kac sites were detected in 841 proteins (Supplementary Table S5), an almost 3-fold increase in coverage of the Kac sites when compared to SILAC enrichment. Of the proteins quantified by MaxQuant, 498 proteins were found to have Kac sites only during hypochlorite stress whereas 11 proteins had Kac sites only during the mock treatment (Figure 6a). The protein with the highest mass spectral count of Kac peptides was the 2Fe-2S ferredoxin HVO_2995 (Supplementary Table S5). This protein showed a 1.5-fold reduction in Kac peptides detected after hypochlorite treatment. 3D modeling of the ferredoxin (Figure 6b) revealed the Kac sites to be near the 2Fe-2S cluster potentially impacting redox potential. In addition to this ferredoxin, other proteins related to central metabolism were shown to be impacted by the hypochlorite treatment including glycerol kinase with a 1.3-fold increase and an acyl CoA synthase with a 4.3-fold decrease in the Kac peptides detected.

When comparing both the label-free datasets with the SILAC dataset, a total of 897 proteins were found to be lysine acetylated across all three datasets with 260 shared between the label-free, LM08, and RC04 (roughly a third overlap). In terms of unique Kac proteins, 474 were detected exclusively in the label-free data whereas 26 and 12 were found to be distinct to the LM08 and RC04 datasets, respectively. Among the proteins detected uniquely in the label-free, many were related to several metabolic processes including macromolecule catabolic, nucleotide metabolic, and cellular protein metabolic processes. This comparison highlights the sensitivity of label-free methods for detecting Kac sites. While the SILAC was not as sensitive, we were able to obtain a quantifiable abundance ratio for determining Kac site changes. 

### 3.5. Lysine Acetylation Sites of H. volcanii Compared to the Related Species H. mediterranei

The SILAC and LFQ datasets of LM08 and RC04 were combined to generate a road map of the *H. volcanii* lysine acetylome (Supplementary Table S6). A total of 1868 lysine residues were found to be acetylated across 897 proteins. The Kac-modified proteins ranged from 1 to 16 Kac sites and averaged 2.1 Kac sites or 1 Kac site per 237 amino acid residues. By comparison, the lysine acetylome of the related *H. mediterranei* detected after growth on glucose-rich medium [16] is observed at 1017 Kac sites among 642 proteins, averaging 1.6 Kac sites with a range of 1 to 10 Kac sites per modified protein. Twelve *H. volcanii* proteins were found modified at ≥1 Kac sites per 28 residues including the coiled-coil protein HVO_0880 and 50S ribosomal protein L44e (HVO_0701), homologous to *H. mediterranei* HFX_0854 and HFX_0660 also found highly Kac-modified [16]. Of the *H. volcanii* Kac proteins, 684 had homologs in *H. mediterranei* (based on UniRef 50) with 311 of these commonly modified by Kac in both species. Protein homologs that were found distinctly Kac-modified in *H. volcanii* or *H. mediterranei* numbered 311 and 146, respectively. Notable examples included heavy Kac modification of the *H. volcanii* glycerol-3-phosphate dehydrogenase (GPDH) subunits GldA1 (6 Kac sites of HVO_1538), GldB1 (2 Kac sites of HVO_1539) and GldC1 (3 Kac sites of HVO_1540) compared to only 1 Kac modification site identified for the *H. mediterranei* GPDH subunit (GldB1, HFX_0792). The differences in carbon source (glycerol vs. complex glucose media) likely account for these findings and suggest that Kac modification activates GPDH, as this enzyme is central to glycerol metabolism [27].

### 3.6. Haloferax Lysine Acetylation and Sampylation Sites

Crosstalk between lysine acetylation and ubiquitination controls vital cellular processes in eukaryotes [28] suggesting similar types of functions may occur in *archaea*. To address this possibility, the *H. volcanii* lysine acetylome was compared to proteins previously found sampylated (ubiquitin-like modified) or bound to ubiquitin-like SAMP proteins in an E1-dependent manner [29,30,31,32,33,34] (Supplementary Table S6). Of the 78 sampylation sites mapped to 60 proteins, approximately one-third (24 sites, 19 proteins) were found common to the *H. volcanii* lysine acetylome. In other words, 24 lysine residues on 19 proteins are targets of sampylation and acetylation in *H. volcanii*. The genomic comparison revealed a large subset of these proteins had homologs in *H. mediterranei* that were also Kac-modified (12 proteins). Four proteins that were sampylated and Kac-modified in *H. volcanii* were related to *H. mediterranei* proteins that were not found in Kac-modified. In addition, three sampylated proteins, which were not Kac-modified in *H. volcanii*, were related to *H. mediterranei* proteins that were Kac-modified. These distinctions may reflect the different growth conditions and/or methods used to detect the Kac sites between the two species. Of the 254 *H. volcanii* proteins that do not have sampylation sites mapped but are found to bind the sampylome in an E1-dependent manner, a high percentage (40–60%) were found lysine acetylated in *H. volcanii* (152 proteins) and/or were related to Kac-modified homologs of *H. mediterranei* (99 proteins). This list included 86 proteins associated with the sampylome that were lysine acetylated in both *Haloferax* species. These results reveal that sampylation and lysine acetylation overlap in modification sites and suggest crosstalk between these PTMs may be conserved in *archaea*.

## 4. Discussion

After exposure to hypochlorite, a clear increase in the abundance of Kac proteins is observed in the *H. volcanii* parent and *∆sir2* mutant strains, establishing a direct link between oxidative stress and lysine acetylation. Other examples linking lysine acetylation to oxidative stress response exist in eukaryotes and to a lesser degree in bacteria [35,36,37,38,39,40,41]; therefore, encountering this direct link in *archaea* suggests fundamental conservation among all three domains of life. However, few, if any, reports exist directly linking lysine acetylation in *archaea* to ROS response. Further exploration of the role of this pathway to oxidative stress in *archaea* can provide a balanced evolutionary perspective of PTMs across all domains of life.

Quantitative analyses using SILAC proteomics provide a powerful tool to compare changes in the proteome during different physiological conditions. However, when comparing to a previously generated SILAC dataset, we did not obtain as deep of coverage of the theoretical proteome (35% compared to 64%) [12]. In that study, which was not focused on the detection of Kac sites, strong cation exchange chromatography (SCX) was performed to increase proteome coverage and reduce sample complexity. However, this step results in sample loss [42] which complicates downstream analysis of lysine acetylation since Kac peptide enrichment requires a high amount of starting material (10–20 mg of protein lysate) [43]. Nevertheless, the total protein dataset provides a snapshot of the alterations in the proteome during hypochlorite stress to facilitate the determination of the occupancy of Kac sites on proteins vs. shifts in protein abundance.

When comparing the total proteomes of LM08 and RC04, a dramatic shift occurs in protein abundance with 253 proteins being downregulated in RC04 whereas on LM08 only 11 proteins were downregulated and 63 upregulated. Lysine acetylation in eukaryotes has sometimes been linked to protein degradation via proteasomes where the acetylated lysine can increase binding affinity for E3-containing complexes, therefore, promoting ubiquitination [44]. In other cases, the acetylated lysine can lead to complex dissociation and, as a result, the leftover components can be degraded by protein degradation machinery as has been shown with HSP90 in eukaryotic cells [45]. Sampylation, an archaeal ubiquitin-like modification system [29,30], has been linked to proteasomal degradation by the 20S proteasome in *H. volcanii* [46]. In previous studies, oxidative stress has been shown to stimulate an increase in archaeal ubiquitin-like sampylation in the presence of DMSO [32] and the small archaeal modifier protein (SAMP1) was shown to increase in abundance during oxidative stress in our previous SILAC dataset. Since the absence of *sir2* promotes the increased abundance of lysine acetylated proteins, this increase can be linked to higher protein turnover. While we were not able to detect any of the SAMPs in our dataset, we did not see any significant change in the levels of the 20S proteasomal core particle or ATPase subunits detected. 

In terms of proteins affected, some proteins upregulated in LM08 were downregulated in RC04. The most notable were those involved in sulfur metabolism pathways (HVO_1031 and HVO_2997). NADPH-dependent thioredoxin reductases transfer electrons to active sites in thioredoxins to reduce protein disulfides and other substrates [47]. This type of protein dithiol/disulfide relay is a common mechanism for oxidative stress defense across all domains of life. In haloarchaea, sulfhydryl groups in cysteine may be used for the mobilization of sulfur to essential sulfur-containing molecules, this is supported by increased levels of cysteine synthase and cysteine desulfurase in *H. volcanii* during oxidative stress as well as cysteine biosynthesis transcripts and thioredoxin transcript increases in *Halobacterium salinarum* [12,48,49]. Among the other proteins downregulated during hypochlorite stress, a pathway enrichment of cobalamin biosynthesis proteins is observed. Cobalamins (e.g., vitamin B_12_) are required as cofactors for a variety of enzymes and are linked to the regulation of transcription factors, binding and posttranscriptional regulation of RNAs, lipid biosynthesis, and amino acid metabolism [50,51]. Moreover, a decrease in proteins related to cell division apparatus assembly was also detected. In Eukaryotes, lysine acetylation of histones has been linked with the regulation of cell cycle and cell division [52]. In bacteria, the *S. enterica* Pat protein was demonstrated to acetylate RcsB, a global regulator involved in cell division [53]. Additionally, pathway enrichment is observed in poryphyrin and tetrapyrrole biosynthetic process which is associated with enzymes involved in the synthesis of carotenoids which serve as important ROS scavengers in halophilic organisms [11]. Finally, in RC04 all predicted enzymes related to DNA topology in *H. volcanii* were shown to be downregulated in abundance. Several examples exist of lysine acetylation of type I DNA topoisomerases in other organisms. In *E. coli*, proteomics studies have shown TopA (DNA topoisomerase I) to be lysine acetylated in residues 13, 45, 346, and 488 which potentially impacts interaction with DNA [54,55]. Additionally, deletion of lysine deacetylase CobB was shown to increase negative supercoiling of DNA as a result of decreased activity of TopA; the mutant also has a slow growth phenotype [56]. Moreover, the deletion of *topA* results in cells being sensitive to oxidative stress [57]. Similarly, in yeast, deletion of *sir2* also affects DNA topology with increased accumulation of negative supercoiling [58]. Our results suggest an association between DNA topology and lysine acetylation is also likely in *H. volcanii*. Overall, these impacts in the proteome of a *∆sir2* mutant can imply a potential challenge during hypochlorite stress in the strain, suggesting an important function of Sir2 for this response. While a transposon mutant of *sir2* was shown to be hyper tolerant [19], transposon insertions can influence the expression of adjacent genes and/or other mutations may have occurred on the genome.

In terms of lysine acetylated protein enrichment, we observed a significant enrichment of DNA topological change networks, acetyl-CoA biosynthesis, and enzymes involved in translation. Of note, all 5 enzymes in the DNA topological change network had acetylation sites detected in the dataset, suggesting DNA topology may be regulated by lysine acetylation. Acetyl-CoA synthetases are the most common lysine acetylation target across all domains of life [17,59,60,61]. Similarly, lysine acetylated proteins in the ribosome and translation apparatus have also been reported in bacteria, eukaryotes, and *archaea*, suggesting fundamental regulatory roles of lysine acetylation in protein translation [16,62,63].

When observing changes in acetylation levels of the detected sites, we noted a significant downregulation of Kac of K914 of GltB (HVO_0869) a glutamate synthase (ferredoxin) EC 1.4.7.1 homolog in the parent strain LM08. These types of enzymes function as glutamate:ferredoxin oxidoreductases using glutamate and oxidized ferredoxin to form glutamine, oxoglutarate, and reduced ferredoxin [64]. Ferredoxin (HVO_2995) was also found to be acetylated at 3 sites: K97, K113, and K119 with K97 increasing in lysine acetylation levels after hypochlorite treatment of LM08. This result suggests that lysine acetylation may regulate ferredoxin and glutamate synthase in maintaining the redox balance of the cell. Other downregulated acetylation sites in LM08 include the Lon protease (HVO_0783). In haloarchaea, Lon proteases have been associated with the biosynthesis of carotenoids that are known to assist with ROS scavenging as a first line of defense [65].

To obtain a more comprehensive perspective on lysine acetylation, the SILAC and LFQ datasets were combined resulting in more coverage of Kac sites. Of note, the most detected acetylated protein was 2Fe-2S ferredoxin (HVO_2995) with the K119 site found to decrease in Kac abundance 1.5-fold during oxidative stress. A homolog of this 2Fe-2S ferredoxin in *Halobacterium salinarum* was the first protein reported to be lysine acetylated in *archaea* [66]. The position of the acetylated lysine residue is conserved in other haloarchaea [67], including *H. volcanii*. The proximity of the acetylation site to the iron-sulfur cluster may have implications to the redox potential. Additionally, decreased Kac modification of glutamate synthase, as observed in the SILAC dataset, can suggest alterations to the protein that may facilitate maintaining the redox state of the cell. While we were able to detect the lysine acetylation of many proteins in both the LFQ and SILAC datasets, significant changes in lysine acetylation state were missed in the SILAC data highlighting limitations of using the SILAC method for accurate quantification of lysine acetylation. In contrast, using a label-free approach while giving a greater coverage of Kac peptides, a true quantitative value of acetylation level was not obtained. Nevertheless, combination of the two methods resulted in providing more complete insights into the lysine acetylome of *H. volcanii* during oxidative stress.

## 5. Conclusions

Our study established a direct link between oxidative stress response and the PTM of lysine acetylation. We find that exposure to sodium hypochlorite yields an increase in Kac targets in the parent strain. This accumulation of Kac targets is more dramatic when examined by immunoblotting analysis in a lysine deacetylase *∆sir2* mutant. Using a quantitative SILAC approach, we examined the impact of the accumulation of lysine acetylation on the proteome of *H. volcanii*. Deletion of *sir2* resulted in a dramatic downregulation of proteins after hypochlorite exposure including those associated with sulfur metabolism, cobalamin synthesis, division septum assembly, and DNA topological change. Using immunoaffinity enrichment, we were able to determine Kac targets during oxidative stress in a quantitative way and observed pathways of DNA topological change, central metabolism, and translation to be regulated. Finally, we used label-free quantification to make up for limitations in our SILAC enrichment and further identified lysine acetylation targets during oxidative stress. However, limitations exist to this approach as we were unable to determine site occupancy of lysine acetylation for a more robust stoichiometric measurement. Furthermore, due to the requirement for a large amount of protein material to perform immunoaffinity enrichment, we had to sacrifice proteome coverage to obtain this lysine acetylome. Optimization of these bottlenecks in analysis can yield a more time-efficient, cost-effective, and comprehensive analysis of lysine acetylation and other PTMs in other organisms. Overall, our results demonstrate new insights into ROS response in halophilic organisms.

## Figures and Tables

**Table 1:** List of strains, plasmids and primers used in this study.

Strain, Plasmid or Primer	Description	Source or Ref.
Strains:		
*E. coli*		
TOP10	F^−^ *mcrA Δ*(*mrr-hsdRMS*-*mcrBC*) Φ80*lacZΔ*M15 *ΔlacX74 recA1 araD139 Δ*(*ara leu*) 7697 *galU galK rpsL* (Str^r^) *endA1 nupG λ*-	Invitrogen
GM2163	F^−^ *ara-14 leuB6 fhuA31 lacY1 tsx78 glnV44 galK2 galT22 mcrA dcm-6 hisG4 rfbD1 rpsL136 dam13*::Tn*9 xylA5 mtl-1 thi-1 mcrB1 hsdR2*	New England Biolabs
*H. volcanii*		
DS70	DS2 cured of plasmid pHV2	[20]
H26	DS70 *∆pyrE2*	[20]
JM503	H26 *∆sir2*	This study
JM506	H26 *∆pat1 ∆pat2*	This study
LM08	H26 *∆lysA ∆argH*	[12]
RC04	LM08 *∆sir2*	This study
Plasmids:		
pTA131	Ap^r^; pBluescript II containing P*fdx-pyrE2*	[20]
pJAM202c	Ap^r^; Nv^r^; pJAM202-derived control	[21]
pJAM4009	Ap^r^; pTA131 carries *pat1* and ~500 bp flanking sequence (pre-deletion plasmid)	This study
pJAM4010	Ap^r^; pTA131 carries *pat2* and ~500 bp flanking sequence (pre-deletion plasmid)	This study
pJAM4011	Ap^r^; pTA131 carries *sir2* and ~500 bp flanking sequence (pre-deletion plasmid)	This study
pJAM4013	Ap^r^; pJAM4009 *Δpat1* (deletion plasmid)	This study
pJAM4014	Ap^r^; pJAM4010 *Δpat2* (deletion plasmid)	This study
pJAM4015	Ap^r^; pJAM4011 *Δsir2* (deletion plasmid)	This study
Primers:		
1756_500_BamHI	5′ ATCGGATCCGCGTTGCCGAGGTAGAAGAACGTC 3′	This study
1756_500_HindIII	5′ TTTAAGCTTCGAACGCGGACTGAGCGCCTCGGA 3′	This study
1821_500_BamHI	5′ TTTGGATCCGGACTCGTCTGTCATACCGCGGGC 3′	This study
1821_500_HindIII	5′ TTTAAGCTTCGCGCCCGCTCTCTATCGACCTCG 3′	This study
KO_HVO_1756R	5′ GGCTCCAGCTTGCCCCTCGGCTTTCGGTG 3′	This study
KO_HVO_1756F	5′ ACCCGCAGTTCGAAAAGTGACCGGACTCCCGGCGAACCT 3′	This study
KO_HVO_1821R	5′ GGCTCCAGCTTGCGCCGCCCTCGTCGTCCGGCG 3′	This study
KO_HVO_1821F	5′ ACCCGCAGTTCGAAAAGT GAGGGCGGCGGCGAGCC 3′	This study
BamHI-HVO_2194	5′ TTCGGATCCCCTCGTCGGGCCACTCGTCC 3′	This study
KpnI-HVO_2194	5′ TTGGTACCCTCCGAACTCCGATAGCGGGCGACCG 3′	This study
delta_sir2_RV	5′ AGCCCAACGGACGGGCCAGCC 3′	This study
delta_sir2_FW	5′ GCGCGACCGGTGCCGCC 3′	This study
hvo_2194_Check_FW	5′ GAGGGAGGTCGGGCACCTGCG 3′	This study
hvo_2194_Check_RV	5′ATTCGATGTCGCCGGAAACGCGG 3′	This study

**Table 2:** Network-based interaction enrichment analysis of proteins of decreased abundance in the *∆sir2* mutant RC04 after hypochlorite stress. Top 10 networks with the greatest number of hits strength and FDR are listed.

Pathway	Detected ^a^	Background ^b^	Strength ^c^	FDR ^d^
DNA topological change	5	5	1.19	0.0026
Division septum assembly	3	3	1.19	0.0479
Cobalamin biosynthetic process	9	17	0.91	0.00032
Purine ribonucleoside monophosphate biosynthetic process	10	23	0.83	0.00039
Tetrapyrrole biosynthetic process	13	30	0.82	2.71 × 10^−5^
DNA-dependent DNA replication	6	14	0.82	0.013
Chromosome organization	13	37	0.73	0.00016
DNA conformation change	10	29	0.73	0.0014
Ribonucleoside monophosphate biosynthetic process	12	36	0.71	0.00044
Pigment biosynthetic process	7	21	0.71	0.016

^a^ Proteins observed in dataset annotated with GO biological process term. ^b^ Predicted total proteins in genome annotated with GO biological process term. ^c^ Log10(observed/expected). ^d^ Shown are *p*-values corrected for multiple testing within each category using the Benjamini–Hochberg procedure.

## Data Availability

Raw mass spectrometry files were deposited to the Proteome Xchange Consortium via the PRIDE [68] partner repository with the dataset identifier PXD041799 (Username: reviewer_pxd041799@ebi.ac.uk, Password: UagMTTQU). Strains generated in this study are available upon request.

## Supplementary Materials

The following supporting information can be downloaded at: https://www.mdpi.com/article/10.3390/antiox12061203/s1, Table S1: List of proteins detected in *H. volcanii* strains LM08 (A) and RC04 (B) by SILAC LC-MS/MS analysis; Table S2: Interaction network enrichment analysis of decreasing proteins in RC04 strain; Table S3: List of acetylated peptides detected in *H. volcanii* LM08 (A) and RC04 (B) strains by SILAC LC-MS/MS analysis; Table S4: Interaction network enrichment analysis of acetylated proteins detected in *H. volcanii* LM08 (A) and RC04 (B) strains; Table S5: Full list of acetylated proteins detected in *H. volcanii* by label-free quantitative (LFQ) LC-MS/MS analysis; Table S6: Comparison of Kac modification within the *H. volcanii* SILAC and LFQ datasets, between the *H. mediterranei* lysine acetylome, and with the *H. volcanii* sampylome.
